# Hypoxia in the St. Lawrence Estuary: How a Coding Error Led to the Belief that “Physics Controls Spatial Patterns”

**DOI:** 10.1371/journal.pone.0138858

**Published:** 2015-09-23

**Authors:** Daniel Bourgault, Frédéric Cyr

**Affiliations:** 1 Institut des Sciences de la Mer de Rimouski, Rimouski, Québec, Canada; 2 Royal Netherlands Institute for Sea Research (NIOZ), Texel, The Netherlands; Auckland University of Technology, NEW ZEALAND

## Abstract

Two fundamental sign errors were found in a computer code used for studying the oxygen minimum zone (OMZ) and hypoxia in the Estuary and Gulf of St. Lawrence. These errors invalidate the conclusions drawn from the model, and call into question a proposed mechanism for generating OMZ that challenges classical understanding. The study in question is being cited frequently, leading the discipline in the wrong direction.

## Introduction

Based on the results of an idealized dissolved oxygen numerical model of the Estuary and Gulf of St. Lawrence, Lefort (2011) [1] and Lefort et al. (2012) [2] arrived at a conclusion that challenges previously published views of oxygen minimum zones (OMZ) and hypoxia in coastal waters. They concluded that the OMZ in the Gulf of St. Lawrence and hypoxia in the St. Lawrence Estuary are primarily controlled by the flow over the variable bathymetry, rather than by the decrease with depth of oxygen consumption by marine organisms, as is commonly argued [3–5]. This led them to conclude that “physics controls spatial patterns”. By that they argue that it is the variable bathymetry that is responsible for the creation of the OMZ and that this OMZ would not otherwise exist if the bottom of the Laurentian Channel were flat.

**Fig 1:**
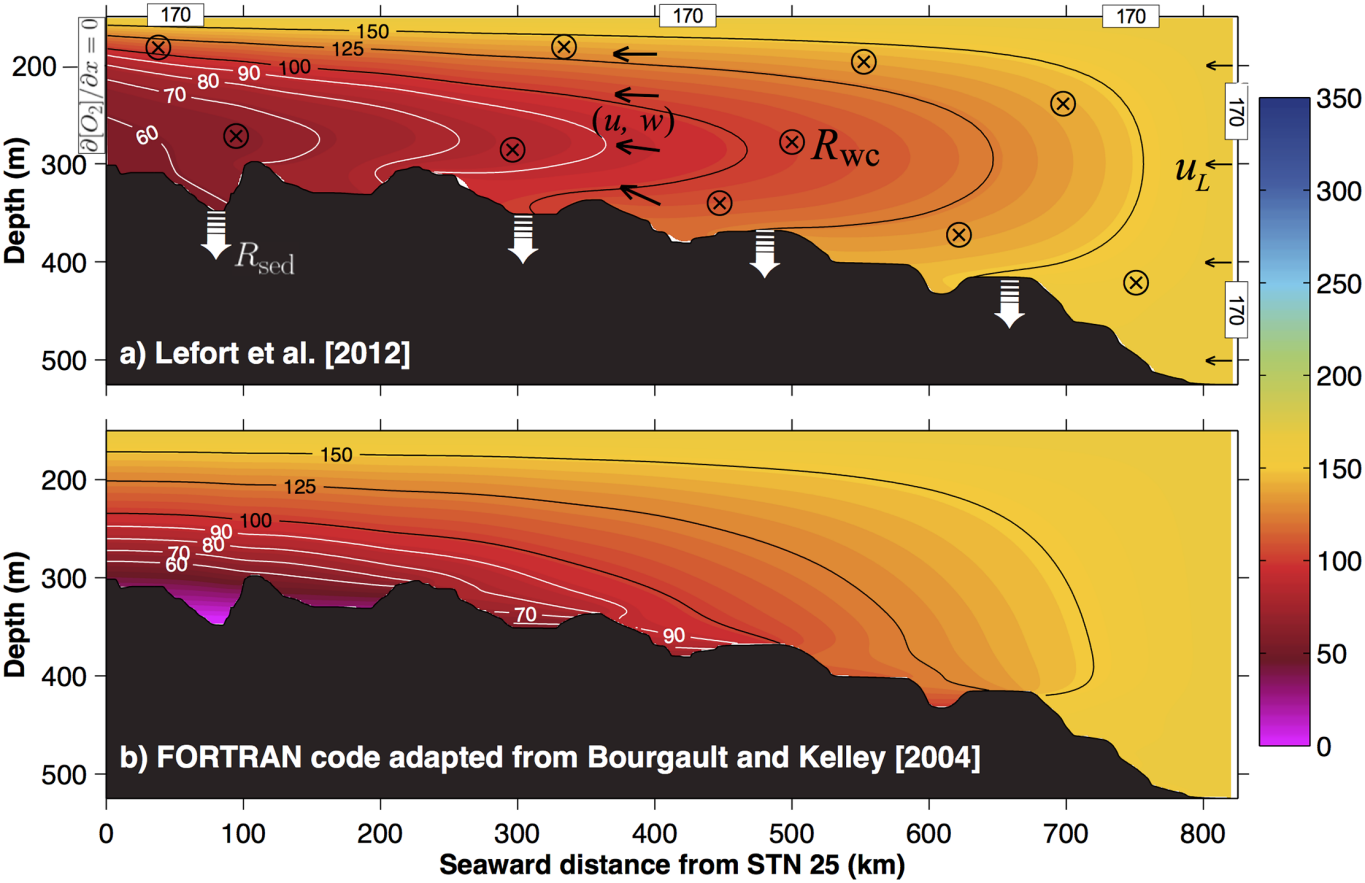
Dissolved oxygen field (colored contours in mmol m^−3^) along the bottom water mass (depth > 150 m) of the Laurentian Channel (a) as simulated by Lefort et al. (2012) [2] (same as their Fig 8) based on the incorrect Eq 3, compared to results obtained using (b) the model of Bourgault and Kelley (2004) [6] based on correctly solving Eq 1. The colorscale is saturated at 0 but the bottom panel reaches unrealistic negative concentrations (−4 mmol m ^−3^). The top panel also schematically shows the boundary conditions (170, ∂ [O_2_]/∂x = 0 and *R*_sed_), constant pelagic respiration (*R*_wc_) schematized throughout with the ⊗ symbol and the circulation (*u*,*w*) proposed by Lefort et al. (2012) [2] (see text for details).

Their conclusion is surprising because their modeled oxygen field (Fig 1a), although showing remarkable similarities with observations, appeared to us both incompatible and unreproducible with the equations, boundary conditions, and parameters that they stated to be using. After several discussions with the authors and mutual sharing of computer codes, we have come to identify simple, yet fundamental, sign errors in their code that invalidate their conclusions. The authors have been informed and have confirmed the unfortunate error.

We present here what the results of their intended model should have been and demonstrate that their model, as posed, is inappropriate for explaining the OMZ in the Gulf of St. Lawrence or hypoxia in the St. Lawrence Estuary.

## Analysis and Results

### The solution of Lefort (2011) and Lefort et al. (2012)

The numerical model developed by Lefort (2011) [1] and Lefort et al. (2012) [2] was intended to solve the following two-dimensional, steady, advection-diffusion equation for dissolved oxygen concentration [O_2_] of the bottom water mass along the Laurentian Channel,

$$u\frac{\partial [O_{2}]}{\partial x}+w\frac{\partial [O_{2}]}{\partial z}=K_{x}\frac{\partial^{2}\left[O_{2}\right]}{\partial x^{2}}+K_{z}\frac{\partial^{2}\left[O_{2}\right]}{\partial z^{2}}+R_{wc}$$ (1)

where *x* is the longitudinal axis positive seaward, *z* is the vertical axis positive upward with its origin at the sea surface, and (*u*,*w*) and (*K*_*x*_,*K*_*z*_) the corresponding velocities and constant eddy diffusivities. *R*_wc_ is a sink term (< 0) for pelagic respiration taken as temporally and spatially constant throughout the domain.

The domain under consideration is the deep water (*z* ≤ *z*_0_ = −150 m) of the Laurentian Channel from Tadoussac to Cabot Strait with a total length *L* = 825km and variable thickness *H*(*x*) (Fig 1). The top boundary, at *z* = *z*_0_, is rigid. Dissolved oxygen concentration is fixed throughout the seaward boundary as well as at *z* = *z*_0_, i.e. [O_2_](*L*, *z*) = 170 mmol m^−3^ and [O_2_](*x*, *z*_0_) = 170 mmol m^−3^. A Neumann boundary condition ∂[O_2_](0, *z*)/∂x = 0 was intended to be imposed at the landward open boundary. A constant benthic oxygen uptake *R*_sed_ was intended to be taken into account as a bottom boundary condition. Their reference run uses: *K*_*x*_ = 1600 m^2^ s^−1^, *K*_*z*_ = 10^−4^ m^2^ s^−1^, *R*_wc_ = −19.6 mmol m^−3^ yr^−1^ and *R*_sed_ = −3540 mmol m^−2^ yr^−1^.

The velocity field (*u*, *w*) was prescribed using the continuity equation

$$\frac{\partial u}{\partial x}+\frac{\partial w}{\partial z}=0$$ (2)

and by fixing a vertically-uniform constant inflow (*u*_*L*_ = −0.003 m s^−1^) at the seaward open boundary, which is equivalent to setting an irrotational potential flow throughout the domain. This point about the circulation becomes important when attempting to understand and interpret the model results.

It is worth pointing out that, with those parameters, the horizontal diffusion term is very high. It is comparable in magnitude to the advection term, i.e. $UL/K_{x}\sim O(1)$, and is about an order of magnitude greater than the vertical diffusion term, i.e. $K_{x}H^{2}/(K_{z}L^{2})\sim O(10)$, where *U* ∼ 10 ^−3^ m s^−1^, *L* ∼ 10^6^ m, *H* ∼ 10^2^ m, *K*_x_ ∼ 10^3^ m^2^ s^-1^, and *K*_z_ ∼ 10^-4^ m^2^ s^-1^.

Lefort (2011) [1] and Lefort et al. (2012) [2] intended to solve Eq 1 for [O_2_] using the Matlab partial differential equation toolbox. Their main result is shown in Fig 1a. Given the prescribed circulation and intended model setup, it is puzzling to see such a pronounced OMZ being produced so close to the seaward boundary where the oxygen concentration is prescribed to be vertically uniform. It is also puzzling that the minimum does not coincide with the region where oxygen is withdrawn at the maximum rate, i.e. near the bottom. The explanation provided [1, 2] is that the oxygen minimum is displaced upward by the vertical velocity near sloping topography (see their discussion at paragraph 27 [2]). This is not a plausible mechanism because the irrotational circulation ensures that the flow must follow the topography: a water parcel near the bottom must remain near the bottom in its journey from the deep gulf into the shallower lower estuary.

The discovered error was that their code was actually solving the following equation, in which the signs of the diffusive terms and fluxes were reversed

$$u\frac{\partial [O_{2}]}{\partial x}+w\frac{\partial [O_{2}]}{\partial z}=-K_{x}\frac{\partial^{2}\left[O_{2}\right]}{\partial x^{2}}-K_{z}\frac{\partial^{2}\left[O_{2}\right]}{\partial z^{2}}+R_{\text{wc}}$$ (3)

These sign errors appear explicitly in Lefort (2011) [1] (her Equation A5).

There are other apparent problems in their results, possibly related to the sign errors discussed above or to other problems in the model implementation. For example, with a Newmann boundary condition at the landward boundary (*x* = 0), one would expect the lines of equal O_2_ concentration to be horizontally flat at the boundary, whereas their resulting field shows horizontal gradients at that boundary (Fig 1a). Another issue is that their oxygen budget presented in their Fig 10, upon which they build their interpretation for the existence of an OMZ, does not balance to zero when integrated either around the top or the bottom closed regions. In steady state, the normal flux integrated across the enclosing area should add up to zero. Yet, the integrated budget in the top region, taking into consideration the pelagic sink, adds up to +12 × 10^10^ mol yr^-1^ (calculated from their Fig 10). Considering that the volume of this top region is 1 × 10^13^ m^3^ (i.e. layer width = 97.5 km, layer thickness = 125 m and layer length = 820 km) indicates that this layer sees its oxygen concentration increasing by 12 mmol m^-3^ every year. This is impossible in the context of a steady-state model and therefore indicates a misinterpretation of the model results.

## The Corrected Solution

These signs errors have important consequences on their results and conclusions. When correctly solving Eq 1 with the proposed boundary conditions and parameters, the resulting oxygen field is markedly different than the one they presented. This comparison is shown in Fig 1a and 1b. Note that for solving Eq 1 we have adapted here a *z*-grid finite difference ocean model that has been well tested and used in many oceanic applications [6].

The corrected solution of Eq 1 (Fig 1b) has the minimum oxygen concentration sitting at the bottom throughout most of the Laurentian Channel, as one would expect from their boundary conditions, benthic oxygen uptake, and prescribed circulation (irrotational potential flow). In contrast, the Lefort et al. (2012) [2] model produces an incomprehensible prominent mid-depth OMZ that happened to match observations.

Note that there are few places in the new simulation where the minimum oxygen concentration lies slightly above the bottom. This can be seen on the seaward side of the topographic bumps located near kilometres 375, 500 and 675 (Fig 1b). These inversions are caused by the high horizontal diffusion used (see the scaling in the previous section) over the varying bottom topography. They only appear on the seaward sides of the bumps because the prominent horizontal gradient is positive (i.e. the overall oxygen concentration increases seaward). In other words, the seaward sides of topographic bumps are somewhat protected from receiving poorly oxygenated water of landward origin from horizontal diffusion but, inversely, can well receive highly oxygenated water of seaward origin. A sensitivity test showed that increasing the horizontal diffusivity exacerbates this effect, while decreasing it diminishes it.

Note also that with this model (Fig 1b), the oxygen concentration drops below zero near the bottom of the head of the Laurentian Channel. In other words, the corrected solution to the problem as defined by Lefort et al. (2012) [2] produces unrealistic results.

## Discussion

In the light of this finding, the interpretation, discussion, and analyses that Lefort (2011) [1] and Lefort et al. (2012) [2] made from their erroneous model results are invalidated (e.g., their oxygen budget, the role of physics *vs.* biogeochemistry, the role of variable bathymetry, their explanation for the existence of the OMZ, etc.).

There is currently no indication that the oxygen spatial pattern in the Gulf of St. Lawrence is principally governed by the local bathymetry. More likely, the mid-depth OMZ observed is predominantly advected from the North Atlantic, where it is produced by mechanisms classically understood [3–5]. This is, for example, supported by oxygen measurements collected by an Argo float that was deployed at the mouth of the Laurentian Channel and that crossed the Gulf Stream [7].

It is important that this negative result be presented because the work of Lefort (2011) [1] and Lefort et al. (2012) [2] is increasingly being used and cited [8–14] and is leading research on hypoxia in the wrong direction. For example, Villate et al., (2013) [9] concluded that “Our results, in agreement with those reported by Lefort et al. (2012) for the St. Lawrence estuary, suggest that the physics of the system and the oxygenic conditions of the source water were mostly responsible for the oxygen depletion and its distribution pattern in the deep waters over the studied period.” Clearly, this sort of element of discussion needs to be reconsidered now that it is known that the results of Lefort et al. (2012) [2] are invalid.

## Conclusion

The equation, boundary conditions, and parameters, proposed by Lefort (2011) [1] and Lefort et al. (2012) [2] are inappropriate when solved correctly for explaining the observed oxygen field and hypoxia in the St. Lawrence Estuary. It is by unfortunate chance that their unrealistic Eq 2 combined with their proposed boundary conditions, parameters and numerical scheme produced remarkable but puzzling agreement with observations. Hypoxia in the St. Lawrence Estuary and the OMZ in the Gulf of St. Lawrence are important features to reproduce correctly with proper theory, and the community must not be left continuing to believe that their model succeeded in reproducing them.

## Supporting Information

S1 Data A Matlab datafile named S1_Data is provided as supporting information. This file contains the following two data structures:

Lefort_et_al =

O2: [821x377 double]

x: [1x822 double]

z: [1x377 double]


Bourgault_Cyr =

O2: [826x201 double]

x: [1x826 double]

z: [1x201 double]


where the structures Lefort_et_al and Bourgault_Cyr contain, respectively, the Lefort et al. (2012) [2] model results (Fig 1a) and the new results presented here (Fig 1b). Each structure contains the oxygen concentration field .O2 (mmol m^-3^), the along-channel axis .x (m) and the vertical axis .z (m).
(MAT)
